# Incidence of Facial Nerve Paralysis After Parotidectomy: Our Experience

**DOI:** 10.7759/cureus.55045

**Published:** 2024-02-27

**Authors:** Estefanie S Otanez, Tatiana Fernandez Trokhimtchouk, Fernando Semanate, Christian Palacios

**Affiliations:** 1 General Surgery, Universidad Internacional del Ecuador, Quito, ECU; 2 Surgical Oncology, Hospital de Especialidades Carlos Andrade Marin, Quito, ECU; 3 Head and Neck Surgery, Hospital de Especialidades Carlos Andrade Marin, Quito, ECU

**Keywords:** facial nerve paralysis, parotid surgery, salivary gland surgery, salivary gland tumor, parotid gland tumor

## Abstract

Facial nerve palsy (FNP) is a well-recognized complication following parotidectomy, with varying reported incidence rates in the literature. Understanding the incidence and factors contributing to FNP is crucial for optimizing patient care and surgical outcomes. A retrospective analysis was conducted on 78 patients who underwent parotidectomy at a tertiary care institution (Hospital de Especialidades Carlos Andrade Marin, Quito) over a 36-month period. Demographic data, preoperative pathology reports, surgical details, and postoperative outcomes, including FNP incidence and severity, were analyzed. The mean age of the cohort was 53 years, with a male-to-female ratio of 0.8:1. Fine needle aspiration revealed benign pathology in 70.5% of cases, with superficial parotidectomy being the most common surgical approach (84.6%). Postoperatively, FNP was observed in 51.2% of cases, with transient paralysis in 62.5% and persistent paralysis in 37.5%. The majority of FNP cases were classified as grade II and III according to the House-Brackmann grading system. A tumor size larger than 4 cm was associated with a higher incidence of FNP (57.5%). This study provides valuable insights into the incidence and severity of FNP following parotidectomy. Despite efforts to standardize surgical techniques, persistent paralysis remains a significant concern.

## Introduction

Salivary gland tumors (SGT) represent a relatively infrequent pathology, with an estimated incidence of 2.5 - 3.0 cases per 100,000 individuals. Among these tumors, the majority are benign, predominantly affecting the major glands [1]. Notably, the parotid gland is the most commonly affected site, accounting for approximately 80% of all SGT cases. Within the parotid gland, approximately 80% of tumors are benign, with pleomorphic adenomas being the most prevalent histological subtype [2]. 

The mainstay of treatment is parotidectomy, a procedure that is commonly performed and generally yields favorable outcomes. Surgical options include superficial, subtotal, and total parotidectomy. With a low recurrence rate of approximately 2%, parotidectomy is effective in addressing the pathology of these tumors [3]. However, a significant drawback of this procedure is the risk of facial nerve palsy (FNP), which can have profound functional and cosmetic implications for patients. 

Rates of FNP reported in the literature exhibit significant variability, spanning from 0%, particularly in cases of long-term palsy, to as high as 80% in the immediate postoperative period. Among the five branches of the facial nerve, the marginal mandibular branch is the most frequently affected. Dysfunction of this branch manifests as altered depression of the commissure and lower lip, leading to asymmetry and distortion of facial expression [3-5].

Various risk factors have been identified as contributors to the occurrence of FNP after parotidectomy, with many related to the surgical technique itself. These factors include neural elongation, stress-strain, compression, traction, heat injury, and operating time. Additionally, certain patient-related factors such as anatomical variations, tumor size, malignancy, and preoperative facial nerve dysfunction have been implicated [6,7]. Given the potential impact of FNP on patient outcomes, it is imperative that patients undergoing parotidectomy are properly counseled on the possibility of this complication.

In our institution, a tertiary center (Hospital de Especialidades Carlos Andrade Marin, Quito) renowned for its expertise in Head and Neck surgery, the department performs approximately 480 surgeries annually, including a significant number of parotidectomies. To contribute to the existing body of knowledge and better understand the incidence of FNP following this procedure, we conducted a comprehensive analysis of all parotidectomies performed from January 2018 to December 2021. By leveraging our institutional data, we aimed to provide valuable insights into the occurrence of FNP and its associated factors, ultimately informing clinical practice and optimizing patient care.

## Materials and methods

This retrospective study analyzed data from all parotidectomy procedures performed by the Head and Neck Surgery Department at our tertiary care institution over a 36-month period, from January 2018 to December 2021. A total of 78 patients were included in the study cohort, encompassing both benign and malignant conditions to ensure a comprehensive representation of parotid gland pathologies. Surgical procedures were performed by five experienced surgeons from the department.

Patients who underwent either superficial or total parotidectomy were included in the study, while cases of enucleation procedures were excluded. Comprehensive data collection included demographic information such as age and gender, along with preoperative pathology reports from fine needle aspiration and documentation of laterality; descriptive statistics were used to characterize the study cohort. 

During surgery, meticulous attention was paid to the technique, adhering to a standardized protocol established by the department. The surgical approach involved a modified Blair incision, with careful dissection and identification of anatomical landmarks for facial nerve trunk localization, including the tragal pointer, tympanomastoid suture, posterior belly of digastric muscle, styloid process, and retromandibular vein. Dissection of the facial nerve was performed in a centrifugal fashion to identify all branches and mobilize parotid parenchyma effectively, with the use of bipolar cautery.

Following the European Salivary Gland Society classification of parotidectomies [8], resections were categorized as superficial parotidectomies when involving levels I and II. In cases where deep lobe resection was indicated, it was performed in a piecemeal fashion to achieve excision of levels III, IV, and V. Following specimen resection, a suction drain was placed, and the wound was closed.

Postoperative outcomes, including FNP, were assessed using the House-Brackmann grading system on day one and during follow-up at six months postoperatively [9]. Patients with preoperative facial palsy were excluded. Pathology reports provided histological details of the excised specimens, including tumor characteristics such as the greater diameter of the tumor. Additionally, the appearance of salivary fistula and Frey syndrome were recorded to capture postoperative complications comprehensively.

The incidence of postoperative FNP was calculated as the frequency of cases presenting with paralysis out of the total number of parotidectomy procedures performed. Subsequently, the incidence rate was determined as the proportion of cases with FNP relative to the total study population, expressed as a percentage. This allowed for the quantification and comparison of FNP occurrence within different patient subgroups based on tumor pathology, size, and other relevant variables.

## Results

This study included a total of 78 patients who underwent parotidectomy, with 36 (46.1%) male and 42 (53.9%) female participants. The mean age of the cohort was 53 years old, ranging from 11 to 94 years. Fine needle aspiration was performed preoperatively on all patients, with 55 (70.5%) individuals receiving a benign pathology report, while 18 (23%) were diagnosed with malignancy, and 5 (6.4%) had inconclusive reports. The majority of patients underwent superficial parotidectomy (66, 84.6%), while 12 (15.4%) underwent total parotidectomy (Table 1). The predominant side for surgery was the right side, accounting for 42 (53.9%) of cases compared to 36 (46.1%) for the left side. During surgery, 10 patients (12.8%) were found to have compromised the main facial nerve trunk or branches due to tumor involvement. The tumor size varied among the study cohort, with 12 patients (15.4%) presenting tumors less than 2 cm in greater diameter, 28 patients (35.9%) with tumors equal to 2 cm and less than 4 cm, and 38 patients (48.7%) with tumors equal to or larger than 4 cm.

**Table 1:** Type of surgery and facial nerve palsy

Type of surgery	n	%	Facial Nerve Palsy	House-Brackmann grading system			
				Grade II	Grade III	Grade IV	Grade V
Superficial parotidectomy	66	84.6%	31	12	12	6	1
Total parotidectomy	12	15.4%	9	2	2	5	
Total	78	100%	40 (51.2%)	14	14	11	1

On final pathology, 59 (75.6%) patients were reported to have benign pathology, 15 (19.2%) malignancy, and 4 (5.2%) patients were found to have conserved histology. Among those with benign pathology, 40 had adenomas, 35 pleomorphic, and 5 polymorphic, 7 were reported as Warthin’s tumor, 3 oncocytomas, 3 myoepitheliomas, 2 lipomas, 2 follicular hyperplasias, 1 duct cyst, and 1 chronic sialadenitis. The cases with a malignant report included seven pleomorphic adenocarcinomas, three metastases from other head and neck malignancies, two cystic adenoid carcinomas, one mucoepidermoid carcinoma, one salivary duct carcinoma, and one primary extraosseous plasmacytoma of the parotid gland (Table 2).

**Table 2:** Types of tumors

Type of tumor		n=78
Benign	Pleomorphic Adenoma	35
	Warthin´s tumor	7
	Pleomorphic Adenoma	5
	Oncocytomas	3
	Myoepitheliomas	3
	Lipomas	2
	Follicular hyperplasias	2
	Duct cyst	1
	Chronic sialadenitis	1
	Conserved histology	4
Total		63
Malignant	Pleomorphic adenocarcinomas	7
	Metastases	3
	Cystic adenoid carcinomas	2
	Mucoepidermoid carcinoma	1
	Salivary duct carcinoma	1
	Extraosseous plasmacytoma	1
Total		15

Patients with preoperative FNP were excluded from the study. Postoperative palsy was detected in 40 of the 78 cases (incidence rate=51.2%) on postoperative day one. Upon six-month follow-up, 25 patients had fully recovered facial motility, while 15 had persistent palsy. According to the House-Brackmann grading system, most FNP cases were classified as grade II (14) and III (14), with 11 cases graded as grade IV and just 1 case graded as grade V. Out of the 14 cases with grade II palsy, only one had persistent alteration on six-month follow-up, while five cases with grade III palsy showed persistence of motility alteration. Most cases with grade IV paralysis resulted in permanent impairment, with only three cases showing signs of recovery (Table 3).

**Table 3:** Facial nerve palsy and recovery FNP: Facial nerve palsy

House-Brackmann grading system	Facial Nerve Palsy	Recovery of FNP	Persistent FNP
II	14	13	1
III	14	9	5
IV	11	3	8
V	1	-	1
Total	40 (51.2%)	25 (62.5%)	15 (37.5%)

Among the 59 patients with benign pathology, 28 had signs of FNP in the postoperative period, including 9 with grade II palsy, of whom 8 fully recovered, 11 with grade III palsy, 7 recovering facial motility, and 7 with grade IV palsy, of whom only 2 recovered facial nerve function. One patient with benign pathology presented with grade V palsy, which did not recover. Among the 15 patients with malignant final pathology, 11 had FNP, including 4 with grade II palsy, all of whom fully recovered, 3 with grade III palsy, with one patient not recovering, and 4 with grade IV palsy, with only one patient recovering. Additionally, one out of the 4 patients with conserved histology developed grade II palsy and fully recovered on follow-up.

**Figure 1:**
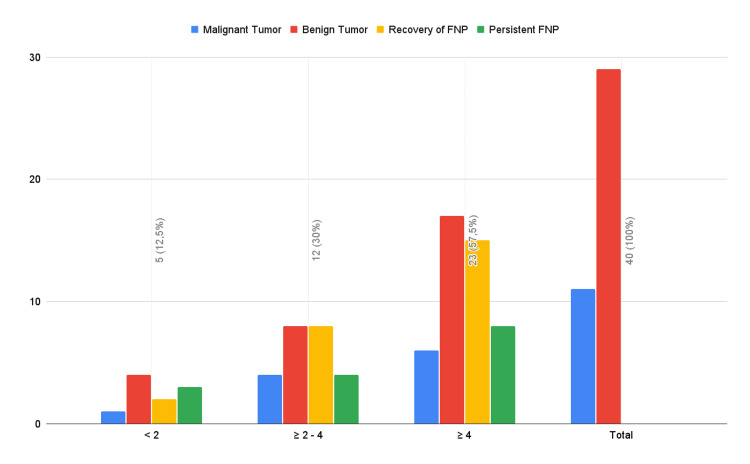
Tumor size and facial nerve palsy FNP: Facial nerve palsy

When considering the tumor size, out of the five patients with a greater diameter of less than 2 cm, 1 presented grade II palsy, 1 grade III palsy without recovery of function, and three cases of grade IV palsy with 2 recovering. Twelve patients with tumors ranging from 2 to 4 cm presented with palsy, 4 with grade II, 5 with grade III, and 3 with grade IV, with 2 out of each of the latter groups not recovering. As for cases with tumors larger than 4 cm, 23 presented with postoperative palsy 9 grade II, 8 grade III, 5 grade IV, and 1 grade V, out of which 8 did not recover function (1, 2, 4, and 1, respectively) (Figure 1).

Regarding complications, only 1 case out of 78 (incidence 1.28%) presented salivary fistula. None reported Frey Syndrome.

## Discussion

The findings of this study provide valuable insights into the occurrence of FNP following parotidectomy, a procedure commonly performed for both benign and malignant salivary gland tumors. Our cohort had a mean age of 53 years, which is similar to the report by Ruas et al. with a mean of 54.2 years, although our male-to-female ratio differed slightly from their findings (0.8:1 vs. 1:1) [10].

Our analysis revealed an overall incidence rate of 51.2% for postoperative FNP, with transient paralysis observed in 62.5% of those cases and persistent paralysis in 37.5% of cases. These findings align with the considerable variability reported in the literature regarding FNP rates following parotidectomy. For example, Infante-Cossio et al. conducted a prospective and descriptive study involving 79 patients undergoing only superficial parotidectomy for pleomorphic adenomas, with 77.2% of patients presenting with FNP, all of whom recovered within 12 months [11]. In contrast, other studies, such as the recent one by Kumar et al. [6], reported transient palsy in 35% of cases and permanent palsy in only 1.66%, although this cohort comprised 60 patients with benign pathology undergoing superficial parotidectomy. These discrepancies underscore the multifactorial nature of FNP occurrence and highlight the importance of considering various patient and surgical factors when interpreting incidence rates across studies. 

Another relevant study by Sethi et al. [12] reported an incidence of 26.7% for temporary weakness and 2.6% for permanent, but again they only included benign pathology and attempted to identify associations with postoperative FNP, it is noteworthy to mention that none of the clinical or pathological variables analyzed were found to be significant risk factors. This observation underscores the crucial role of the surgical technique in influencing post-operative outcomes. Specifically, meticulous and atraumatic handling of and dissection around the facial nerve during parotid surgery are considered the most important factors in minimizing the risk of FNP. This emphasizes the significance of surgical skill and technique in achieving optimal patient outcomes and underscores the importance of ongoing training and adherence to standardized protocols in parotid surgery.

The predominance of transient FNP in our cohort aligns with previous studies highlighting the reversible nature of most postoperative facial nerve injuries. However, it is noteworthy that a significant proportion of patients experienced persistent paralysis, which is the most relevant complication after parotid tumor surgery and can have profound functional and cosmetic implications. The distribution of FNP severity, as assessed by the House-Brackmann grading system, further underscores the spectrum of outcomes observed in our study, with the majority of cases classified as grades II and III [13].

Several factors may contribute to the occurrence of FNP following parotidectomy, including surgical technique, tumor characteristics, and patient-related factors. In our study, meticulous attention was paid to the surgical technique, with standardized protocols aimed at minimizing nerve injury. Compromised facial nerve branches were identified intraoperatively in 12.8% of cases. Tumor characteristics, such as size and histological subtype, may also influence the risk of FNP. While the majority of tumors in our cohort were benign, including pleomorphic adenomas and Warthin's tumors, a larger tumor size (more than 4 cm) presented a higher incidence of FNP (57.5%). This finding is consistent with other studies suggesting that increased tumor size correlates with greater surgical complexity and a higher likelihood of nerve injury [14].

To address the need for a more in-depth study on this topic, future research efforts could focus on several key areas. Firstly, conducting prospective studies with larger sample sizes would enhance the generalizability of findings and provide more robust evidence regarding the incidence and predictors of FNP following parotidectomy. Additionally, longitudinal studies could track patient outcomes over an extended period to evaluate the long-term effects of FNP on functional and cosmetic aspects, thereby providing valuable insights for patient counseling and management.

Our study contributes to the existing body of knowledge on FNP following parotidectomy and underscores the importance of tailored surgical approaches and vigilant postoperative monitoring to optimize patient outcomes. A major limitation is the lack of intraoperative nerve monitoring in our institution, a method that has shown good outcomes regarding the prevention of nerve injury. Intraoperative nerve monitoring allows for real-time assessment of nerve function and identification of potential areas of injury, enabling surgeons to modify their technique and minimize the risk of nerve damage [15,16]. Its absence in our study may have influenced the incidence and severity of postoperative FNP, highlighting the need for its incorporation into future surgical protocols to further enhance patient safety and outcomes. 

Moreover, a comprehensive analysis of patient-reported outcomes and quality-of-life measures following parotidectomy would provide a holistic understanding of the impact of FNP on patients' well-being. This could involve incorporating validated instruments to assess facial function, aesthetic outcomes, and psychosocial aspects, thereby addressing the broader implications of FNP beyond clinical parameters [17].

## Conclusions

In conclusion, our study highlights the high incidence of FNP following parotidectomy, with transient paralysis observed in the majority of cases. Despite efforts to standardize surgical techniques and minimize nerve injury, persistent paralysis remains a significant complication with potential long-term implications for patient quality of life. In the clinical setting, comprehensive preoperative counseling and diligent postoperative care are essential to adequately prepare patients for the possibility of facial nerve dysfunction and to optimize their overall experience and recovery following parotidectomy.
